# Agglutination and hemolytic crossmatching to determine transfusion reaction differences between large and small breed goats

**DOI:** 10.1111/jvim.16738

**Published:** 2023-05-25

**Authors:** Cileah M. Kretsch, Flavio H. Alonso, Maggie Buktenica, Meera C. Heller

**Affiliations:** ^1^ Department of Medicine and Epidemiology, School of Veterinary Medicine University of California, Davis, 1 Garrod Drive Davis California 95616 USA; ^2^ Veterinary Medical Teaching Hospital, School of Veterinary Medicine University of California, Davis, 1 Garrod Drive Davis California 95616 USA

**Keywords:** anemia, blood groups, caprine, hemolysis, hemorrhage

## Abstract

**Background:**

Blood transfusions are performed frequently in goats, but crossmatches are rarely performed.

**Hypothesis/Objectives:**

Determine differences in the frequency of agglutination and hemolytic crossmatch reactions between large and small breed goats.

**Animals:**

Healthy adult goats, 10 large and 10 small breed.

**Methods:**

Two hundred eighty major and minor agglutination and hemolytic crossmatches: 90 large breed donor to large breed recipient (L‐L), 90 small breed donor to small breed recipient (S‐S), 100 large breed donor to small breed recipient (L‐S). A linear mixed model with treatment group (L‐L, S‐S, L‐S) as a fixed effect and individual crossmatch as a random effect was used to identify variations in reaction frequency among groups and individuals.

**Results:**

Frequency of major agglutination reactions for L‐L, S‐S, and L‐S were 3/90 (3.3%), 7/90 (7.8%), and 10/100 (10.0%), respectively. Frequency of major hemolytic reactions for L‐L, S‐S, and L‐S were 27/84 (32.1%), 7/72 (9.7%), and 31/71 (43.7%). Individual pairings and groupings had no effect on agglutination reactions. Individual pairings had no effect on the frequency of hemolytic reactions. For major hemolytic crossmatches, pairwise comparisons identified higher frequencies of reactions when comparing L‐L to S‐S (*P =* .007) and L‐S to S‐S (*P <* .001).

**Conclusion and Clinical Importance:**

Goats experience increased frequencies of hemolytic reactions compared to agglutination. Significant increases in hemolysis were seen between large breed donors and small breed recipients, compared to small breed pairings. Additional studies are required to determine correlations between crossmatches and transfusion reactions.

**Abbreviations**
CAE: caprine arthritis and encephalitis virus
CL: caseous lymphadenitis
EDTA: ethylene‐diaminetetraacetic acid
kgs: kilograms
PCV: packed cell volume
RBC: red blood cell
VMTH: William R. Pritchard Veterinary Medical Teaching Hospital
WAD: West African Dwarf

## 1 INTRODUCTION

Whole blood transfusions are used to treat anemia attributed to various conditions in goats, including severe parasitism, trauma, and hemolytic anemia.^1^, ^2^, ^3^ Crossmatches are performed rarely before the administration of whole blood because of a large number of blood groups in small ruminants combined with few choices for donor animals, expense, lack of expertise, and lack of established protocols. Goats have at least 6 known blood group systems (A, B, C, E, F, and R), with the B system containing over 52 factors.^2^ Consequently, blood typing is rarely performed, and the frequency of first‐time transfusion reactions is considered relatively low. A recent retrospective study reported a transfusion reaction frequency of 15.6% for small ruminants, but risk related to size and breed was not evaluated.^3^

The published maximum amount of blood that a small ruminant donor can provide is 10‐15 mL/kg of body weight or 20% of total blood volume per month.^1^ Many small breed goats (Nigerian Dwarf, Pygmy) can donate a maximum of 300‐450 mL of blood, based on an average weight of 30 kg.^1^ However, a large breed goat (Alpine, Boer, Nubian, Saanen, La Mancha) can donate 750‐1125 mL of blood at a time based on an average weight of 75 kg.^1^ Consequently, large breed goats have more utility as blood donors than do smaller breeds, especially if being kept as blood donors at a clinic.

Unlike small animals, ruminants are more prone to hemolytic reactions compared to agglutination reactions.^2^ Therefore, performing both an agglutination and hemolytic crossmatch is recommended when screening donors for a given recipient.^2^ No published protocols are available for agglutination or hemolytic crossmatches in small ruminants, and reports have not been published concerning the frequency of crossmatch incompatibility among goats of variable size and breed. Our objectives were to develop a crossmatch protocol for goats and determine the differences in frequency of agglutination and hemolytic crossmatch reactions among goats of similar and different sized breeds. We hypothesized that an increased frequency of crossmatch reactions would occur between large and small breed goats compared to crossmatches between goats of the same breed size, and that goats will have a higher frequency of hemolytic reactions as compared to agglutination reactions.

## 2 MATERIALS AND METHODS

### 2.1 Animals

Twenty goats divided equally into large and small breeds were included. A physical examination was performed on each goat before blood collection and enrollment in the study. Only goats considered healthy based on physical examination and medical history were included in the study. In addition, goats that were difficult to handle for blood collection and any goat that required >1 needle insertion or redirection were excluded to minimize the occurrence of sample hemolysis. The 10 large breed goats consisted of 5 Saanens, 2 Alpines, and 3 LaManchas. These goats were owned by the University of California, Davis, Animal Science Department. The 10 small breed goats consisted of 5 Nigerian Dwarf and 5 Pygmy goats that were privately owned. Of the 20, 17 were open females (14 currently lactating), 1 wether, and 2 bucks. All animals were routinely vaccinated for *Clostridium tetani* and *C. perfringens* type C and D and were sero‐negative for caprine arthritis and encephalitis virus, caseous lymphadenitis, and *Mycobacterium avium paratuberculosis* based on farm protocols. The study protocol was approved by the Animal Care and Use Committee at the University of California, Davis.

### 2.2 Study design

Thirty milliliters of blood were drawn from the jugular vein of each goat, using 20‐gauge vacutainer needles, and placed in 2 red top (additive free) tubes and 2 lavender top tubes containing ethylenediaminetetraacetic acid (EDTA). The EDTA‐containing samples were inverted slowly to promote homogenization and prevent clot formation. To minimize hemolysis, samples were allowed to cool to room temperature for 30‐60 minutes before being stored at 4°C for up to 72 hours.

One EDTA tube from each patient was submitted to the William R. Pritchard Veterinary Medical Teaching Hospital (VMTH) Clinical Diagnostic Laboratory for a CBC. Plasma protein concentrations were measured using a hand‐held optic refractometer (ADE Advanced Optics inc., Oregon City, Oregon) for each sample after centrifugation (Unico, Dayton, New Jersey) of a microhematocrit tube at 3000 rpm for 3 minutes. Plasma fibrinogen concentration was determined using the heat precipitation method.^4^ Finally, a Romanowski Wright's‐stained blood smear from each animal was evaluated by a clinical pathologist (FA) to assess for erythrocytic, leukocytic, and thrombocytic morphological abnormalities. These diagnostic tests were performed in the event an individual animal was found to have increased frequencies of crossmatch reactions compared to others. These tests are not a part of the crossmatch protocol.

Each animal was used as both a donor and recipient in crossmatch combinations, as presented in Table 1. A total of 280 major and minor agglutination and hemolytic crossmatches were performed: 90 large breed donor to large breed recipient (L‐L), 90 small breed donor to small breed recipient (S‐S), and 100 large breed donor to small breed recipient (L‐S). The crossmatch procedure was adapted from established protocols used in dogs and horses and is described below.^2^, ^4^, ^5^

**TABLE 1:** Blood crossmatching analysis design for 20 goats based on large (L) and small (S) breed size.

Large breed—small breed (n = 100)									
L1‐S1	L2‐S1	L3‐S1	L4‐S1	L5‐S1	L6‐S1	L7‐S1	L8‐S1	L9‐S1	L10‐S1
L1‐S2	L2‐S2	L3‐S2	L4‐S2	L5‐S2	L6‐S2	L7‐S2	L8‐S2	L9‐S2	L10‐S2
L1‐S3	L2‐S3	L3‐S3	L4‐S3	L5‐S3	L6‐S3	L7‐S3	L8‐S3	L9‐S3	L10‐S3
L1‐S4	L2‐S4	L3‐S4	L4‐S4	L5‐S4	L6‐S4	L7‐S4	L8‐S4	L9‐S4	L10‐S4
L1‐S5	L2‐S5	L3‐S5	L4‐S5	L5‐S5	L6‐S5	L7‐S5	L8‐S5	L9‐S5	L10‐S5
L1‐S6	L2‐S6	L3‐S6	L4‐S6	L5‐S6	L6‐S6	L7‐S6	L8‐S6	L9‐S6	L10‐S6
L1‐S7	L2‐S7	L3‐S7	L4‐S7	L5‐S7	L6‐S7	L7‐S7	L8‐S7	L9‐S7	L10‐S7
L1‐S8	L2‐S8	L3‐S8	L4‐S8	L5‐S8	L6‐S8	L7‐S8	L8‐S8	L9‐S8	L10‐S8
L1‐S9	L2‐S9	L3‐S9	L4‐S9	L5‐S9	L6‐S9	L7‐S9	L8‐S9	L9‐S9	L10‐S9
L1‐S10	L2‐S10	L3‐S10	L4‐S10	L5‐S10	L6‐S10	L7‐S10	L8‐S10	L9‐S10	L10‐S10

*Note*: Example of crossmatch design for large breed donor to small breed recipients with each cell indicating a crossmatch pair with donor‐recipient format. Each goat was assigned a letter‐number combination (L1‐L10, S1‐S10) based on breed size. Groupings include 90 large breed combinations, 90 small breed combinations, and 100 large breed donor to small breed recipient combinations. Large breeds = Saanen, Alpine, La Mancha. Small breed = Nigerian Dwarf and Pygmy.

All agglutination and hemolytic crossmatches were graded by a single clinician (CK) throughout the study. Before the study, this individual was trained by the university's clinical pathology department.

#### 2.2.1 Complement solution

Before the crossmatch procedure, 10 mL of blood in a red top tube was collected from the jugular vein of a university‐owned blood donor goat that was not included in the study. The donor goat was an adult castrated male Nubian. The blood was centrifuged for 5 minutes at 3000 rpm (Fisher Scientific, Waltham, Massachusetts) and serum placed in glass tubes. Centrifugation was repeated 10 times, each time followed by transfer of supernatant into a clean glass tube. A 1:8 dilution of the final supernatant in 0.9% sterile saline (Braun, Irvine, California) was prepared. Commercial rabbit complement (Pel‐Freez Biologicals, Rogers, AR) then was diluted in the prepared diluted goat serum to create a 1:2 dilution of complement: dilute serum. Aliquots were placed in cryogenic tubes (Thomas Scientific, Swedesboro, New Jersey) and frozen at −18°C.

#### 2.2.2 Sample preparation

All donor and recipient red blood cells were washed before the crossmatch procedure. Briefly, 2 mL of whole blood from EDTA tubes and an equal volume of the 0.9% saline solution were placed in glass tubes using plastic transfer pipettes (Cole‐Parmer, Vernin Hills, Illinois), and centrifuged at 3000 rpm for 2 minutes. The supernatant was discarded, and the RBC pellet was resuspended in saline. This washing procedure was repeated 3 times. Finally, a 5% solution of washed RBCs was created by mixing 0.1 mL of washed RBCs with 2.0 mL of 0.9% saline.

Blood from red top tubes was centrifuged at 3000 rpm for 5 minutes and serum was transferred to a clean glass tube and refrigerated at 4°C until needed.

#### 2.2.3 Agglutination crossmatch

**FIGURE 1:**
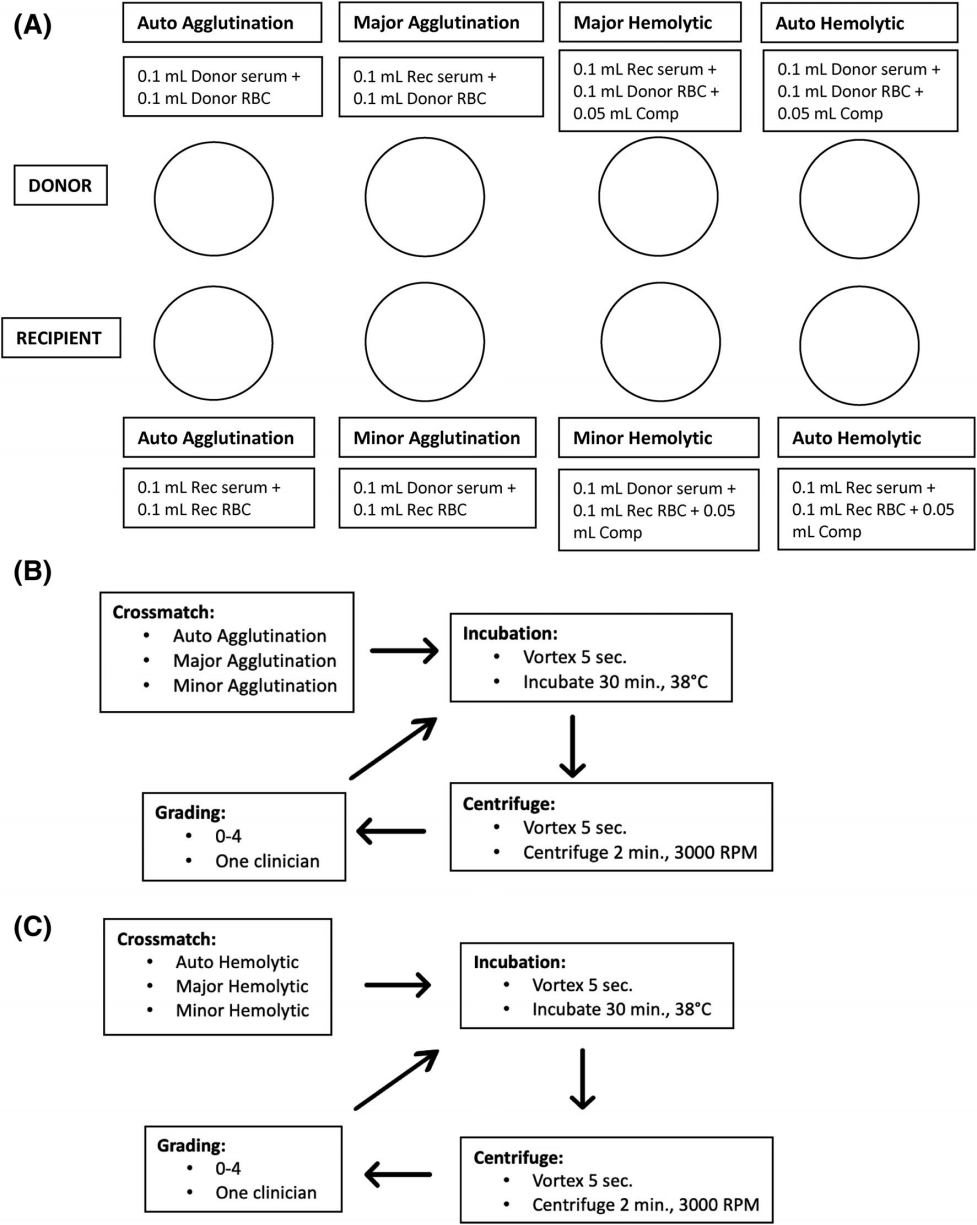
Diagram of agglutination and hemolytic crossmatch setup. (A) Set up of individual test tubes for each crossmatch pair. (B) Flow chart depicting the steps for the agglutination crossmatch protocol. (C) Flow chart depicting the steps for the hemolytic crossmatch protocol. A second incubation and grading were performed for each crossmatch to detect delayed reactions. Comp, 1:2 complement solution; Rec, recipient.

Diagrams of the agglutination and hemolytic crossmatch setups and procedures are depicted in Figure 1. Major, minor, and auto control agglutination crossmatches were performed as follows. As indicated for each reaction, plastic transfer pipettes were used to add serum and washed RBCs to clean glass tubes (Fisherbrand, Hampton, New Hampshire). Each major agglutination tube contained 0.1 mL (2 drops) of recipient serum and an equal volume of donor washed RBCs. Each minor agglutination tube contained 0.1 mL of donor serum and 0.1 mL of recipient washed RBCs. Finally, each auto control contained 0.1 mL of the animal's own serum and washed RBCs. Each tube was vortexed (Axiom, Brooklyn, New York) and then incubated for 30 minutes at 38°C. After incubation, the samples were vortexed for 5 seconds and centrifuged for 2 minutes at 3000 rpm to create an RBC pellet at the bottom of the glass tube.

Each tube was gently agitated under an agglutination lamp (Becton, Dickinson and Company, Parsippany, New Jersey) to observe the stability of the RBC pellet and a grade from 0 to 4 was assigned. Agglutination was graded as follows: 0, the button at the bottom of the tube dissolves rapidly after agitation creating a completely homogenous mixture; 1, separation of the pellet into small pinpoint aggregates with a heterogenous background; 2, separation of the pellet into multiple small buttons and a clear to heterogeneous background; 3, a single large button followed by multiple smaller buttons in a clear background; 4, a tight button of cells remained at the bottom of the tube after substantial agitation.^2^

#### 2.2.4 Hemolytic crossmatch

**FIGURE 2:**
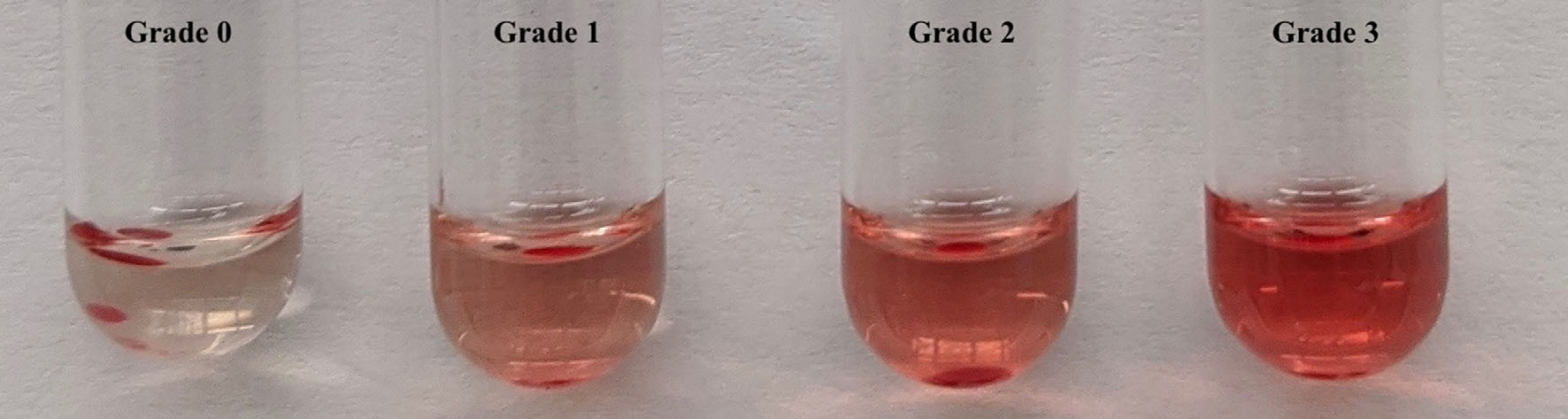
Hemolytic grading score including 0, 1, 2, and 3. Grade 4 not imaged here. A score of 0 was considered negative for hemolysis with a clear background of similar color to the control. A grade of 4 was assigned to samples with a dark red background and considered the most severe. Grades 1‐3 were assigned based on the color gradient.

Major, minor, and auto control hemolytic crossmatches were performed. Each major, minor, and auto control tube contained the same mixture as described above, but 0.05 mL (1 drop) of 1:2 complement was added to each reaction mixture. The samples were vortexed, incubated, and centrifuged as described above. Hemolysis was graded on a scale of 0 to 4 (Figure 2). This scoring system was adopted from the university's crossmatch protocol used in horses. A score of 0 was considered negative for hemolysis with a clear background of similar color to the control. A grade of 4 was assigned to samples with a dark red background and considered the most severe.

#### 2.2.5 Delayed reactions

Incubation and grading were repeated, immediately after initial grading, for each crossmatch, using the same samples to detect any delayed reactions in both the agglutination and hemolytic crossmatches. This second incubation was performed for 30 minutes at 38°C. After incubation, the samples were vortexed for 5 seconds and centrifuged for 2 minutes at 3000 rpm to create an RBC pellet. Grading for both agglutination and hemolytic crossmatches was assigned as described above. Delayed reactions were defined as an increase from grade 0 to ≥1 between the first and second incubations. All results include total reactions from both the first and the second incubation, unless stated otherwise.

### 2.3 Statistical analysis

Data analysis was performed using statistical software (GraphPad Prism v 8.4.3, GraphPad Software, San Diego, California; JMP Pro 16.2, SAS Institute, Cary, North Carolina). Because no published crossmatch data is available for goats, the expected frequency of agglutination and hemolytic crossmatch reactions was set at 20% based on published transfusion reaction data.^3^ Based on this assumption and our goal to determine reaction frequency between similar and different breed groups, 95 crossmatches were needed. Normality of the data points was assessed using the Shapiro‐Wilk test. For parametric data, mean ± SD were reported, whereas median and 95% confidence intervals (95% CI) were reported for nonparametric data.

Crossmatches were graded on a scale of 0‐4, and grades ≥1 were considered a reaction for both hemolytic and agglutination crossmatches. A linear mixed model, with treatment group (L‐L, S‐S, L‐S) as a fixed effect and the individual crossmatch pairs (eg, L1‐L4) as a random effect was used to identify variations in reaction frequency among groups and between individual pairs. As indicated, further pairwise comparisons using Tukey's test were performed to identify specific differences between groups or pairs. A *P* value <.05 was considered significant.

## 3 RESULTS

### 3.1 Descriptive statistics

Mean (±SD) age was 3.0 (±1.2) years. Mean (±SD) heart rate, respiratory rate, and temperature were 94 (±21) beats/minute, 43 (±10) breaths/minute, and 38.7 (±0.4)°C, respectively. Table 2 contains descriptive statistics of the CBC, and plasma protein and fibrinogen concentrations for the large and small groups. Blood smear evaluation identified no evidence of erythrocytic protozoa or bacteria. The most common morphologic changes on blood smears included the presence of dacrocytes (4/20, 20%), acanthocytes (2/20, 10%), schistocytes (2/20, 10%), and slight anisocytosis (2/20, 10%).

**TABLE 2:** Descriptive statistics of (A) erythrogram, (B) leukogram, and (C) platelets, plasma protein, and fibrinogen for 20 goats, divided according to breed size.

(A)						
Group	HCT (%)	Hgb (g/dL)	MCV (fL)	MCH (pgm)	MCHC (g/dL)	RDW (%)
Large	27.08 ± 3.59	9.51 ± 1.09	19.28 ± 2.41	6.78 ± 0.78	35.2 ± 0.80	21.89 ± 1.90
Small	31.57 ± 5.35	10.76 ± 1.82	20.39 ± 1.50	6.97 ± 0.57	34.23 ± 0.42	25.44 ± 2.22
RR	23.00‐36.00	8.20‐12.40	15.0‐23.0	5.50‐8.00	32.50‐38.00	21.00‐28.00

(B)						
Group	WBC (×10^3^/μL)	Neutrophils (×10^3^/μL)	Lymphocytes (×10^3^/μL)	Monocytes (/μL)	Eosinophils (/μL)	Basophils (/μL)
Large	7.92 ± 1.64	3.49 ± 0.88	4.09 ± 0.93	138.9 ± 68.85	114.0 (67.5‐186.0)	44.20 ± 15.7
Small	11.32 ± 2.85	4.85 ± 1.38	5.64 ± 2.73	472.7 ± 237.3	207.0 (135.3‐329.3)	81.2 ± 27.81
RR	5.00‐17.00	0.700‐7.60	2.50‐12.00	70.0‐570.0	0.0‐2000.0	0.0‐250.0

(C)				
Group	Platelets (×10^3^/μL)	MPV (fL)	PP (g/dL)	Fibrinogen (mg/dL)
Large	269.7 ± 192.3	9.41 ± 2.83	7.27 ± 0.38	100 (75‐200)
Small	307.2 ± 246.2	9.91 ± 3.29	8.27 ± 0.64	150 (100‐425)
RR	340.0‐900.0	3.70‐7.10	6.00‐7.50	100‐400

*Note*: For parametric data, mean ± SD were reported, whereas median (95% CI) were reported for nonparametric data. Lab‐specific reference ranges (RR) are provided from the VMTH. Large breeds = Saanen, Alpine, La Mancha. Small breeds = Nigerian Dwarf and Pygmy.

### 3.2 Agglutination

In total, 36/280 crossmatches (12.9%) received an agglutination score ≥1 in all 3 groups. When comparing agglutination reactions between major and minor crossmatches for the L‐L, S‐S, and L‐S groups, 3 of the 6 (50.0%), 7 of the 14 (50.0%), and 10 of the 16 (62.5%) were associated with major crossmatches, respectively. Seven of the 36 reactions (19.4%) were delayed reactions identified after the second incubation. All grades for delayed reactions were scored 1. Further information on agglutination reactions, including the distribution of grades, is summarized in Table 3.

**TABLE 3:** (A) Agglutination and (B) hemolytic crossmatch results for L‐L, S‐S, and L‐S groupings divided by major and minor crossmatches, grade, and incubation.

(A)									
		Incubation 1						Incubation 2	Total (≥1)
		0	1	2	3	4	Total (≥1)	Delayed	
L‐L (90)	Major	88 (97.8%)	1 (1.1%)	1 (1.1%)	0	0	2 (2.2%)	1 (1.1%)	3 (3.3%)
	Minor	88 (97.8%)	1 (1.1%)	1 (1.1%)	0	0	2 (2.2%)	1 (1.1%)	3 (3.3%)
	Total	176 (97.8%)	2 (2.2%)	2 (2.2%)	0	0	4 (4.4%)	2 (2.2%)	6 (6.7%)
S‐S (90)	Major	83 (92.2%)	6 (6.7%)	1 (1.1%)	0	0	7 (7.8%)	0	7 (7.8%)
	Minor	83 (92.2%)	6 (6.7%)	1 (1.1%)	0	0	7 (7.8%)	0	7 (7.8%)
	Total	166 (92.2%)	12 (13.3%)	2 (2.2%)	0	0	14 (15.5%)	0	14 (15.6%)
L‐S (100)	Major	91 (91.0%)	3 (3.0%)	2 (2.0%)	4 (4.0%)	0	9 (9.0%)	1 (1.0%)	10 (10.0%)
	Minor	98 (98.0%)	2 (2.0%)	0	0	0	2 (2.0%)	4 (4.0%)	6 (6.0%)
	Total	189 (94.5%)	5 (5.0%)	2 (2.0%)	4 (4.0%)	0	11 (11.0%)	5 (5.0%)	16 (16.0%)

(B)									
		Incubation 1						Incubation 2	Total (≥1)
		0	1	2	3	4	Total (≥1)	Delayed	
L‐L (84)	Major	68 (81.0%)	12 (14.3%)	4 (4.8%)	0	0	16 (19.1%)	11 (13.1%)	27 (32.1%)
	Minor	66 (78.6%)	14 (16.7%)	4 (4.8%)	0	0	18 (21.5%)	11 (13.1%)	29 (34.5%)
	Total	134 (79.8%)	26 (31.0%)	8 (9.5%)	0	0	34 (40.5%)	22 (26.2%)	56 (66.7%)
S‐S (72)	Major	69 (95.8%)	3 (4.2%)	0	0	0	3 (4.2%)	4 (5.6%)	7 (9.7%)
	Minor	69 (95.8%)	3 (4.2%)	0	0	0	3 (4.2%)	1 (1.4%)	4 (5.6%)
	Total	138 (95.8%)	6 (8.3%)	0	0	0	6 (8.3%)	5 (6.9%)	11 (15.3%)
L‐S (71)	Major	44 (62.0%)	21 (30.0%)	4 (5.6%)	1 (1.4%)	0	26 (37.0%)	5 (7.0%)	31 (43.7%)
	Minor	66 (93.0%)	3 (4.2%)	1 (1.4%)	0	0	4 (5.6%)	0	4 (5.6%)
	Total	110 (77.5%)	24 (33.8%)	5 (7.0%)	1 (1.4%)	0	30 (42.2%)	5 (7.0%)	35 (49.3%)

*Note*: Values in incubation 2 represent delayed reactions with a grade 0 in the first incubation that are now ≥1. Sample size in each group (L‐L, S‐S, L‐S) reflects total crossmatches after removal of pairs with positive autologous controls in the first incubation. Numbers in each cell indicate the number of total agglutination or hemolytic reactions in each group for each category.

Linear mixed model analysis indicated that treatment group (L‐L, S‐S, L‐S) had no effect on the frequency of agglutination reactions for both major (*P =* .2) and minor (*P =* .4) crossmatches. Individual crossmatch pairs similarly had no effect for both major and minor crossmatches (*P >* .99). Further pairwise comparisons were not performed.

### 3.3 Hemolysis

Sample hemolysis can hinder the ability to accurately grade hemolytic reactions.^2^ Consequently, any hemolytic autologous control tube with lysis present during the first incubation and its corresponding cross‐matches were excluded from the study. For example, hemolysis in a hemolytic autocontrol tube for the L1‐L4 pairing meant that all hemolytic crossmatch tubes for that pairing were discarded. Fifty‐three hemolytic crossmatches were discarded. In the L‐L, S‐S, and L‐S groups, 6, 18, and 29 hemolytic crossmatches were excluded, respectively. As a result, 84, 72, and 71 hemolytic crossmatches were included in the data analysis for the L‐L, S‐S, and L‐S groups.

In total, 102/227 crossmatches (44.9%) received a hemolysis score ≥1 with 56/84 (66.7%), 11/72 (15.3%), and 35/71 (49.3%) in the L‐L, S‐S, and L‐S groups, respectively. When comparing hemolytic reactions in major versus minor crossmatches, 27 of the 56 (48.2%), 7 of the 11 (63.6%), and 31 of the 35 (88.6%) were associated with major crossmatches in the L‐L, S‐S, and L‐S groups, respectively. Thirty‐two of the 102 reactions (31.4%) were delayed reactions. A majority of delayed reactions (24/32, 75.0%) increased by 1 grade, and the remainder increased by 2 grades. Further information on the hemolytic reactions, including the distribution of grades, is summarized in Table 3.

Interestingly, unlike in the agglutination crossmatches, a portion of the hemolytic crossmatches displayed delayed hemolysis in the autologous control tubes during the second incubation. In brief, 47/227 (20.7%) crossmatches had this effect, including 16/84 (19.0%), 12/72 (16.7%), and 19/71 (26.7%) in the L‐L, S‐S, and L‐S groups, respectively. When this finding was present, gradings for the crossmatches were based on the first incubation. The data in Table 3B does not include the autologous control tubes that exhibited delayed hemolysis described here.

Linear mixed model analysis indicated that the random variable of individual crossmatch pairs had no effect on the frequency of hemolytic reactions for both major and minor crossmatches (*P >* .99). However, treatment groups were identified to have an effect in both major and minor crossmatches (*P <* .001). In the major hemolytic crossmatches, pairwise comparisons identified significant differences when comparing the L‐L to the S‐S groupings (*P =* .01) and the L‐S to the S‐S grouping (*P <* .001). In the minor hemolytic crossmatches, differences when comparing the L‐L to the L‐S groupings (*P* < 0.001), as well as the L‐L to the S‐S groupings (*P* < 0.001), were identified. In summary, when compared to the S‐S groupings, a significantly increased frequency of hemolytic reactions was identified within the L‐L and the L‐S groupings.

### 3.4 Agglutination versus hemolysis

A higher proportion of hemolytic reactions compared to agglutination reactions was seen in the L‐L and L‐S groupings. In the L‐L group, 66.7% of the reactions were positive for hemolysis compared to 6.7% positive for agglutination. The L‐S group had 49.3% of the reactions positive for hemolysis compared to 16% positive for agglutination. The S‐S group had similar proportions, with 15.3% positive for hemolysis and 15.6% positive for agglutination.

## 4 DISCUSSION

We developed a simple crossmatch protocol for goats. As hypothesized, our study results reinforced that goats, similar to other large animals, have a higher frequency of hemolytic reactions compared to agglutination. Similarly, differences in the frequency of hemolytic crossmatch reactions among breed groups were identified. Most pertinent to the objectives, a higher frequency of major hemolytic reactions occurred when blood from large breed donors was paired with small breed recipients compared to small breed donors paired with small breed recipients. Unexpectedly, the large breed pairings also had increased frequencies of hemolytic reactions compared to small breed pairings.

### 4.1 Agglutination

In our study, 12.9% of the total crossmatches performed were positive for agglutination. Within the L‐L, S‐S, and L‐S groups, 6.7%, 15.6%, and 16.0% of crossmatches showed agglutination, respectively. This finding is similar to a previous study that assessed transfusions in sheep that cited 17.1% incompatible agglutination crossmatches.^6^

### 4.2 Hemolysis

Overall, our results indicate a higher number of hemolytic reactions compared to agglutination reactions. Small ruminants, horses, and cattle RBCs are less likely to agglutinate than the RBCs of dogs and cats. Similarly, most horse alloantibodies are hemolysins rather than hemagglutinins.^2^

Major crossmatches determine if antibodies are present within the recipient's serum against antigens present on the donor's RBC surface, whereas minor crossmatches test for the presence of antibodies in donor serum against the recipient's RBCs. Dogs, cattle, sheep, and goats are considered to have low concentrations of naturally occurring alloantibodies.^2^ Goats have been reported to have a naturally occurring anti‐R antibody and can result in acute reactions when a goat is transfused with R‐positive blood.^2^

The L‐S group had significantly a higher frequency of major hemolytic crossmatch reactions compared to the S‐S group. This finding was not detected with the minor hemolytic crossmatches. Although the reason for the differences identified between the large and small breed goats is unknown, the significantly higher number of reactions within the major crossmatches suggests an increased concentration of alloantibodies in serum of likely naturally occurring origin within the smaller breeds against the erythrocytes of the larger breeds. Interestingly, the L‐L group had similarly high frequencies of positive reactions compared to S‐S in both the major and minor hemolytic crossmatches and increased minor hemolytic reactions compared to the L‐S grouping. All of these findings suggest an increased concentration of alloantibodies within the serum of large breed goats against surface antigens of other large breed goats. The clinical relevance of the repeated finding of increased serum alloantibodies targeted toward the surface antigens of large breed erythrocytes in this study is unclear. These findings could indicate a higher concentration or diversity of naturally occurring alloantibodies in goats than previously has been thought or an increase in R‐positive blood types in large breed goats. However, the reason is difficult to determine without additional studies to assess specific breed differences, blood types, and alloantibodies, as has been done in other species.^5^, ^7^ The importance of these alloantibodies and their clinical correlation with an acute hemolytic transfusion reaction is unknown. Animals with low concentrations of alloantibodies may not always mount a massive acute hemolytic response but rather may slowly upregulate alloantibody production and gradually destroy transfused RBCs over a few days.^5^ In dogs and cats, the antigenicity of certain RBC antigens and their association with acute hemolytic reactions versus delayed destruction of RBCs (if alloantibodies are present) is known.^5^, ^7^

### 4.3 Limitations

The main limitations of our study were sample storage time and sample hemolysis. All samples were used within 72 hours, but some samples were utilized at the start of this period and others at the end. All serum samples were separated and stored in individual glass tubes before the study. Whole blood was stored in EDTA tubes, and aliquots were removed to prepare washed RBCs as needed. In the clinical setting, crossmatches are performed immediately after blood collection, and little information is available on the effect of storage, especially for small ruminants. One study in horses cited poor reproducibility in hemolytic and agglutination crossmatch results with blood that had been stored for 1‐4 weeks.^8^ However, this study did not evaluate storage times <7 days.^8^ A study in humans reported reliable crossmatch results for up to 10 days of storage when erythrocytes were suspended in 3% to 5% saline.^9^ Future studies evaluating the effects of storage time on crossmatch results and reproducibility in goats are warranted.

In our study, 53 autologous control reactions contained signs of hemolysis during the first incubation and were discarded, along with their associated crossmatches. Reasons for hemolysis can include small needle gauge, alcohol residue on the skin, difficult venipuncture, excessive anticoagulant, sample shaking, abrupt changes in temperature, prolonged storage, and excessive centrifugation.^10^ To mitigate these factors, animals were not included in the study if they required >1 needle penetration of the skin or redirection, and 20 gauge needles and vacutainers were utilized for all samples. Samples were allowed to cool to room temperature before refrigeration. Other factors that could not be avoided were high environmental temperatures (>32°C) during the study sampling period and 1‐2 hours of transport to the laboratory.

Similarly, after the second incubation, 47 autologous control tubes contained signs of hemolysis that was not present after the first incubation. Erythrocyte osmotic fragility, a measure of how easily an erythrocyte will lyse in hypotonic solutions, has been studied in goats.^11^ Although in our study washed RBC's initially were placed in an isotonic suspension of 0.9% NaCl and later mixed with serum, there may have been a component of lysis associated with fragility that occurred between incubations. In a study evaluating erythrocyte fragility in goats, bucks were found to have lower percentages of hemolysis compared to dry, lactating, and pregnant does.^11^ Erythrocytes in females are assumed to be more susceptible to osmotic stress because of lipid peroxidation secondary to hyperlipidemia from estrogen.^11^ Consequently, when performing the crossmatch protocol described in our study, sample handling to mitigate hemolysis is crucial. Clinicians may choose to skip the second incubation. Additional studies evaluating hemolytic crossmatch results based on sex are warranted. Similarly, further research assessing differences in RBC fragility over multiple incubations is indicated to determine the true cause of these reactions (ie, hemolysis versus red cell fragility).

Important limitations of the study design include the number of individuals in the study, diversity among groups, the dependent nature of the donor‐recipient pairs, and the potential for clustering from random effects. Although a large number of crossmatches was performed, only 20 individuals were represented and the serum and RBC's of each individual were utilized within and between groups. Similarly, the 20 goats represented different breeds, ages, and both males and females. Although this approach was chosen to be more representative of a clinical setting, these factors could have led to clustering and random effects, causing the variations in crossmatch frequency seen. As a result, a mixed model was used to assess for variations caused by random and fixed effects. However, because of the crossmatch pairings, we were unable to assess the effects of all random effects, including age and sex. However, a study in humans assessing the prevalence of alloantibodies found no correlation between sex or age and the presence of alloantibodies during blood typing and crossmatching.^12^ The only significant relationship correlated with the presence of alloantibodies was prior transfusion history.^12^

No published protocols specifically for crossmatches in ruminants are available. Therefore, no reference standard protocol was available to follow in the development of this study. Consequently, the protocols reported previously in various textbooks, published studies, and at the VMTH were used.^2^, ^4^, ^6^

Finally, all scores were assigned by a single individual who was not blinded to the crossmatch pairings throughout the study. Without blinding, an inherent risk of bias exists because of awareness of crossmatch groupings (L‐L, S‐S, and L‐S) and individual pairings.

The applicability of our findings in a clinical setting when performing blood transfusions is unknown and requires further investigation. Although our study did not identify any individual effect on crossmatch reaction frequencies, it is important to note that our results represent a small population of healthy animals. Compromised patients may be at an increased risk of transfusion reactions. Similarly, prior dystocia and the use of blood‐contaminated needles could lead to development of alloantibodies, potentially increasing the risk for crossmatch and transfusion reactions.

Although we reported the frequency of agglutination and hemolytic crossmatch reactions, it is unknown whether crossmatch scores in goats equate to acute or delayed transfusion reactions, shortened RBC survival, or outcome. Future studies correlating in vitro crossmatch results to in vivo effects are needed. A study assessing transfusion reactions in cats found a larger increase in mean PCV 24 hours post‐transfusion when major crossmatches were performed.^13^ Similarly, RBC survivability based on crossmatch results has been studied in horses, and cross‐match incompatibility was significantly associated with decreased RBC survival leading to an RBC half‐life of 4.7 days versus 33.5 days with compatible crossmatches.^14^ Interestingly, horses transfused with crossmatch incompatible donors developed acute febrile episodes 30 days after transfusion.^14^ Acute intravascular hemolysis with concurrent hemoglobinemia and hemoglobinuria can be seen with incompatible combinations in small animals.^2^ Similarly, delayed reactions can be seen up to 14 days after transfusion, and extravascular hemolysis is more prominent.^2^ Many current publications in small ruminants focus on acute changes in vital parameters during the actual transfusion, with hyperthermia being the most commonly cited acute adverse effect.^3^ However, this reaction is most likely a response to leukocyte or platelet antigens or less commonly bacterial contamination of blood products rather than an erythrocyte antigen‐antibody response.^2^

## 5 CONCLUSIONS

Based on our results, smaller breeds are potentially at increased risk for hemolytic reactions when receiving blood transfused from larger breed goats. However, additional studies involving the correlations among crossmatch score, transfusion reactions, and RBC survival time are required to support this hypothesis. A second incubation of donor's and recipient's washed RBCs and serum seems to be important to increase the sensitivity of a crossmatch reaction in this species.

## CONFLICT OF INTEREST DECLARATION

Authors declare no conflict of interest.

## OFF‐LABEL ANTIMICROBIAL DECLARATION

Authors declare no off‐label use of antimicrobials.

## INSTITUTIONAL ANIMAL CARE AND USE COMMITTEE (IACUC) OR OTHER APPROVAL DECLARATION

Approved by University of California, Davis, IACUC, protocol #21561.

## HUMAN ETHICS APPROVAL DECLARATION

Authors declare human ethics approval was not needed for this study.
